# Toxicity Profile of the Oceanic Pufferfish *Lagocephalus lagocephalus* in the Eastern Atlantic Area

**DOI:** 10.3390/md24060195

**Published:** 2026-06-01

**Authors:** Nathália Nocchi, Álvaro Santana-Mayor, Adrián Conde-Díaz, Víctor Hernández-Lopez, Adriana Rodríguez Hernández, Alberto Brito, Ana R. Díaz-Marrero, José J. Fernández

**Affiliations:** 1 Instituto Universitario de Bio-Orgánica Antonio González (IUBO AG), Universidad de La Laguna (ULL), Avenida Astrofísico Francisco Sánchez 2, 38206 La Laguna, Spain; nathalianocchi@ull.edu.es (N.N.); vhernanl@ull.es (V.H.-L.); 2 Biotecnología Marina, IUBO-ULL, Unidad Asociada al IPNA-CSIC, 38206 La Laguna, Spain; 3 Unidad Departamental de Química Analítica, Departamento de Química, Facultad de Ciencias, Universidad de La Laguna (ULL), Avenida Astrofísico Francisco Sánchez 3, 38206 La Laguna, Spain; asantanm@ull.edu.es (Á.S.-M.); acondedi@ull.edu.es (A.C.-D.); 4 BIOECOMAC (Biodiversidad, Ecología Marina y Conservación), UD Ciencias Marinas, Departamento de Biología Animal, Edafología y Geología, Facultad de Ciencias (Sección Biología), Universidad de La Laguna (ULL), Avenida Astrofísico Francisco Sánchez 3, 38206 La Laguna, Spain; adrianar@ull.edu.es (A.R.H.); abrito@ull.es (A.B.); 5 Instituto de Productos Naturales y Agrobiología (IPNA), Consejo Superior de Investigaciones Científicas (CSIC), Avenida Astrofísico Francisco Sánchez 3, 38206 La Laguna, Spain; 6 Departamento de Química Orgánica, Universidad de La Laguna (ULL), Avenida Astrofísico Francisco Sánchez 3, 38206 La Laguna, Spain

**Keywords:** *Lagocephalus lagocephalus*, Tetraodontidae, marine toxin, tetrodotoxins, saxitoxins

## Abstract

In recent years, the pufferfish *Lagocephalus lagocephalus* has been recorded with unusual frequency in coastal areas of the Canary Islands. The most notable episodes occurred in March and November 2017, when numerous shoals were observed along the coasts of the Western Canary Islands. A toxicological study of these episodes was carried out, analyzing liver, kidney, gonads, skin, and muscle of a representative population. In all toxic samples (33.3% and 41.7% of specimens in March and November 2017, respectively), only the liver extract showed toxicities, using a mouse biological assay (MBA). The toxicological profile was determined by UHPLC-MS-MS, identifying saxitoxin (STX) and tetrodotoxin (TTX) congeners. This analytical methodology was optimized to determine 26 marine toxins. Thus, in the March 2017 episode, the toxicological profile was characterized by the co-occurrence of tetrodotoxins (TTX and 4-epiTTX) and paralytic shellfish toxin (PST) analogues (dcSTX, dcneoSTX, and doSTX); however, STX and neoSTX emerged as the dominant toxins in specimens collected during the November 2017 episode. The results show that *L. lagocephalus* in the Canary Islands presents a variable and dynamic toxicological profile, strongly influenced by environmental factors. These findings highlight the need for continued monitoring and for analytical approaches capable of capturing this complexity and assessing potential risks to public health.

## 1. Introduction

The oceanic puffer *Lagocephalus lagocephalus* (Linnaeus, 1758) (Tetraodontiformes: Tetraodontidae) is a pelagic cosmopolitan species of pufferfish, occurring in tropical and subtropical oceans worldwide [1]. It inhabits waters down to at least 1000 m depth and is rarely found in nearshore environments [2]. No commercial fishery currently targets this species in the Atlantic, and it is considered potentially toxic, as are many members of the family Tetraodontidae. While certain pufferfish species are consumed in Asia under strict regulations, *L. lagocephalus* is not authorized for human consumption, and the commercialization of any member of this family is strictly prohibited in the European Union [3,4,5].

The presence of this specie in the waters of the Canary Islands has been known for quite some time as a consequence of the tropicalization process and with data of sporadic catches between the surface and 600 m depth [6]. In recent years, an increase in records of *Tetraodontiform* species, including *L. lagocephalus*, has been reported in Spanish waters, likely linked to ocean warming and changing oceanographic conditions [7]. However, it was not until the summer of 2004 that a massive spawning aggregation was recorded for the first time on the coast of El Hierro, at shallow depth (less than 20 m). The fish formed numerous and dense shoals at a shallow depth. This episode recurred in 2014 and 2017, also during exceptional oceanographic episodes in concordance with high temperatures. The most important difference in 2017 was that the aggregations were numerous, and they spread throughout the Western Canary Islands (El Hierro, La Palma, La Gomera and Tenerife) [8]. The duration of the spawning process extended in time from June to October, and the fish caused many incidents in professional and recreational fishing operations, with consequent social alarm. Due to the high availability of the fish during these massive aggregations, some recreational fishers harvested the meat for self-consumption; however, no intoxication was reported. Despite not knowing the real toxicity profiles, and in the absence of toxicological data, public information was transmitted about the possible toxicity of this species with the recommendation not to consume it, along with a prohibition on marketing it due to an absence of published toxicological data [9,10].

Due to the fact that many species of the Tetraodontidae family are highly toxic and their consumption has caused serious poisoning episodes in other regions, particularly in the Mediterranean Sea [11,12], a toxicological study of this event in the Canary Islands was carried out as a preventive measure. The occurrence of these toxins is more prevalent in certain species, but may vary depending on factors such as season, reproductive condition, or environmental exposure [3,4,5]. In most cases, toxins are concentrated in internal organs, particularly in the liver and gonads, although they may contaminate muscle tissue during improper handling or cleaning of the fish [3,5,13,14]. Although many tetraodontid species are not commercially targeted, their relative abundance and the potential presence of toxins justify their consideration in toxicological studies [9]. In this context, the organs and tissues of oceanic specimens of this species were examined, and toxicity was assessed using the mouse bioassay (MBA). High-performance liquid chromatography coupled with mass spectrometry (HPLC–MS) was used to characterize the toxin profiles.

## 2. Results

### 2.1. Estimation of Toxin by Mouse Biological Assay (MBA)

All extracts obtained from different organs (liver, kidney, gonads, skin, and muscle; *n* = 15 for March 2017 and *n* = 12 for November 2017) were analyzed using the mouse biological assay (MBA). The detection limit for the MBA was established based on the standardized procedure, where a positive result was defined by the death of at least two out of three mice within 24 h after intraperitoneal injection of the extract. The sensitivity of the assay was consistent with the internationally recognized threshold for total neurotoxicity in fish extracts. Overall, toxicity was detected in between 33% and 42% of the specimens analyzed and was exclusively confined to liver extracts (Table S1). In contrast, kidney, gonads, skin, and muscle extracts showed no detectable toxicity, indicating a strong organ-specific distribution of toxic compounds. Toxic liver extracts were detected in 33.3% of specimens collected in March 2017 and 41.7% of specimens collected in November 2017. Although the prevalence of toxic individuals was similar between sampling events, the highest toxicity values were recorded in November. Marked inter-individual variability was observed. Some specimens exhibited very high tetrodotoxin-equivalent concentrations, whereas others showed low or undetectable toxicity. No clear relationship was found between toxin concentration and fish body size. A detailed summary of MBA results for liver extracts, including death times, is provided in Table S1.

### 2.2. Toxin Profile

**Figure 1:**
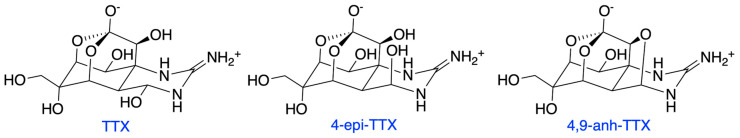
Chemical structure of analyzed TTXs.

In the liver samples, the presence of tetrodototoxin (TTX, *m*/*z* 320) and 4-epi-tetrodotoxin (4-epiTTX, *m*/*z* 320) (Figure 1) was detected.

The qualitative profile presented in Table 1 was conducted on the subset of specimens that exhibited toxicity in the MBA and for which sufficient extract volume remained to complete the UHPLC-MS/MS analytical protocol. Due to the limited amount of tissue in some specimens and the volume consumed during the initial biological screening, chemical characterization was prioritized for four toxic samples from March 2017 and three from November 2017. The qualitative profile (Table 1) reveals a marked seasonal variation in the distribution of studied toxins in *L. lagocephalus* liver extracts. During the March sampling episode, the toxicological profile did not show a clear predominance of a specific toxin group but rather a comparable distribution between tetrodotoxins (TTXs) and paralytic shellfish toxin (PST) analogues. Specifically, TTX and 4-epiTTX were detected in a limited number of samples (two to three specimens), similar to dcSTX, dcneoSTX, and doSTX, which exhibited a comparable occurrence pattern across the dataset. This indicates that both toxin families contributed similarly to the overall profile during this period. M2 analogue was also detected in Specimen 01 among the entire dataset in this period. In contrast, the parent saxitoxins, STX and neoSTX, were not detected in any of the March samples.

**Figure 2:**
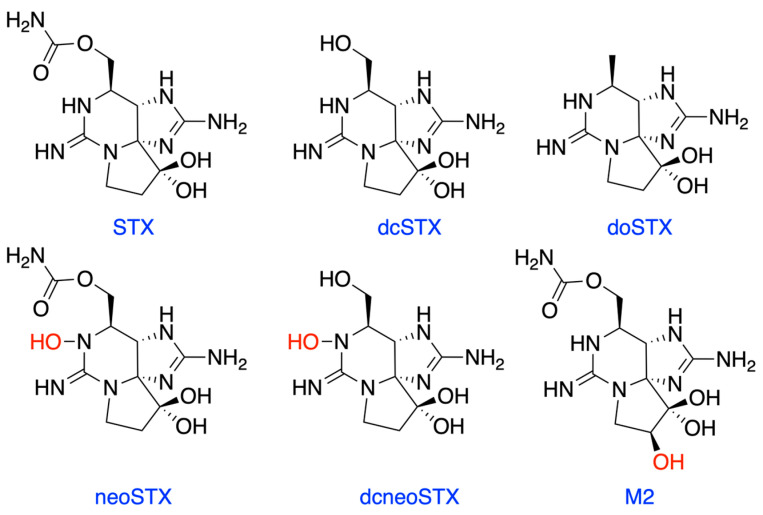
Chemical structure of detected STXs related compounds.

In contrast, a clear shift in toxin profile was observed in the November samples, characterized by an increased prevalence of saxitoxin (STX) and its analogues, collectively referred to as paralytic shellfish toxins (PSTs). STX was consistently detected in all November samples, accompanied by neoSTX, dcSTX, and dcneoSTX, which were also widely present (Figure 2), thereby constituting the dominant toxin group during this period. In contrast, tetrodotoxins were largely absent, with TTX detected in only one sample, while 4-epi-TTX and the M2 analogue were not detected in any of the November specimens. This marked transition highlights a seasonal shift from a mixed toxin profile in March to a PST-dominated profile in November.

Overall, these results demonstrate a seasonal transition from a mixed TTX/PST profile in March, where TTX, 4-epi-TTX, and various PST analogues (dcSTX, dcneoSTX, and doSTX) contributed similarly, to a predominantly PST-rich profile in November, characterized by the marked presence of parent STX and neoSTX. This variation may reflect changes in environmental conditions, dietary sources, or microbial symbionts influencing toxin biosynthesis and accumulation *in L. lagocephalus.*

## 3. Discussion

This study provides the first toxicological assessment of *L. lagocephalus* from the eastern Atlantic, revealing a varied and organ-specific distribution of neurotoxins. Not all specimens showed toxicity; it was only detected in some of the sampled individuals. This pronounced inter-individual variability, characterized by the coexistence of highly toxic and non-toxic individuals, has been widely described in pufferfish and is generally linked to differences in environmental exposure and feeding behavior rather than intrinsic species-specific traits [3,4].

A clear pattern was observed at the tissue level. Toxicity appeared only in liver samples, while the remaining organs analyzed showed no detectable levels. Similar results have been reported in other tetraodontids, where TTXs tend to accumulate mainly in liver and gonads and are generally absent from muscle [3,13]. Although TTX exerts its toxic effects at the neuronal level, its localization in hepatic tissue suggests that the liver functions mainly as a storage and regulation compartment [15]. Experimental studies have shown that pufferfish can selectively accumulate toxins through controlled uptake mechanisms, likely involving specific transport systems that allow sequestration without self-intoxication [16].

The absence of a clear relationship between toxicity levels and fish size further supports the hypothesis that toxin accumulation is environmentally driven. TTX is commonly thought to originate from marine microorganisms and to be transferred through the food web, meaning that individual diet likely plays a key role [3,4,17]. Under this scenario, differences in diet could explain why some individuals accumulate toxins while others do not [3,4]. Consequently, individuals within the same population may exhibit markedly different toxin levels depending on feeding history and habitat conditions, explaining the variability observed.

Some differences between sampling periods were also apparent. Although the proportion of toxic individuals remained similar between sampling periods, higher toxicity values were observed in November. Seasonal variability has been reported in other pufferfish species, and this variability is often observed in associations with, for example, prey availability or temperature [3,16]. In the present study, the exact drivers cannot be identified, but the results suggest that toxin accumulation is not constant over time.

Beyond changes in toxicity levels, differences in toxin composition were also evident. UHPLC–MS/MS analysis revealed that TTXs and PST analogues showed a comparable distribution in March, whereas PSTs, including saxitoxin and related analogues, were more relevant in November. This dual toxin profile highlights the capacity of *L. lagocephalus* to accumulate different classes of neurotoxins depending on environmental conditions. Similar observations have been reported in specimens from the northeastern Atlantic, where PSTs were detected mainly in liver but also in intestines and gonads [14]. Taken together, these results point to a strong influence of trophic exposure and environmental availability of toxin-producing organisms in shaping toxin profiles [16,18]. Overall, this suggests that toxin profiles in this species may be temporally dynamic, reinforcing the importance of considering seasonal factors in toxicological assessments.

The detection of both TTXs and PSTs within the same organism suggests the possibility of co-occurrence of multiple toxin classes. Since both groups act as sodium channel blockers [19], their combined presence may have relevant toxicological implications that are best captured through an integrated assessment. In this context, the implementation of the MBA in this study served as a necessary screening tool to integrate the overall biological risk and neurotoxicity. While instrumental methods like UHPLC-MS/MS offer superior selectivity and sensitivity for identifying and quantifying known toxins, the biological assay remains a critical benchmark for confirming total neurotoxic activity. This is especially relevant for species like *L. lagocephalus*, where the co-occurrence of different toxin families may result in complex toxicological interactions or synergies not fully elucidated by chemical analysis alone [19]. Furthermore, this approach is consistent with scientific research aimed at characterizing emerging hazards in non-commercial species [8], distinguishing it from routine regulatory monitoring of commercial seafood where the transition to animal-free methods is strictly prioritized [9].

For this reason, once the integrated toxicity was confirmed by MBA, the use of UHPLC–MS/MS became particularly important. The proposed analytical method aligns with current strategies for PST determination and provides a high level of selectivity. This is particularly important when dealing with structurally related toxins such as TTXs and PSTs, whose co-occurrence may hinder accurate identification using less selective techniques. The use of a QqQ-MS/MS analyzer operating in multiple reaction monitoring (MRM) mode with two transitions per analyte ensures full compliance with EU confirmatory criteria [20]. In contrast to the large number of methods reported in the literature based on LC with fluorescence detection (LC–FLD), the use of tandem mass spectrometry (LC–MS/MS) in the present work is a significant analytical advantage, particularly in terms of selectivity and unequivocal identification. LC–FLD methods typically require pre- or post-column derivatization and rely on retention time and fluorescence response. In this context, LC–MS/MS operating in MRM mode provides compound-specific transitions, allowing the simultaneous monitoring of precursor and product ions with high selectivity. The use of at least two transitions per analyte further ensures confirmatory identification, significantly reducing the risk of false positives or misidentification. This is especially critical for PSTs and related toxins, whose structural similarity and frequent co-occurrence complicate their discrimination by conventional detection techniques. Therefore, the implementation of LC–MS/MS not only enhances analytical confidence but also enables accurate multi-toxin profiling without the need for derivatization, representing a clear advancement over traditional LC–FLD methodologies [21]. From a chromatographic perspective, the application of a mixed-mode HILIC/weak anion-exchange BEH column provides an effective alternative to the more commonly used BEH Amide or Glycan stationary phases, maintaining comparable retention mechanisms for highly polar toxins while potentially offering improved selectivity for specific analogues [22]. Moreover, the relatively short run time of less than 11.5 min enables high sample throughput, which is particularly advantageous considering the inherent lability of the studied compounds. Given that PSTs and TTXs may undergo degradation or interconversion under unfavorable conditions such as prolonged exposure to non-optimal pH, temperature, or matrix effects, minimizing the analysis time is essential to preserve their native profiles. In this context, the rapid chromatographic separation not only improves laboratory efficiency but also contributes to maintaining analyte integrity, thereby enhancing the reliability and accuracy of the qualitative and quantitative results.

From a public health perspective, these findings should not be overlooked. Tetrodotoxin is highly potent and heat-stable, and even low doses can lead to severe intoxication [4,23]. Although no poisoning cases have been reported in the Canary Islands, studies from nearby Atlantic regions have already documented the presence of PSTs in *L. lagocephalus* [14], suggesting that this species could act as a vector of marine toxins in European waters. Oceanic pufferfish seems to be increasingly reported in Atlantic coasts [7], which may represent a new potential vector of TTX and PSTs in the food web. In addition, the increasing detection of TTX in European waters has raised concern and led to growing regulatory attention [9,10,24], emphasizing the need for monitoring and public awareness.

Overall, the results show that *L. lagocephalus* in the Canary Islands presents a variable and dynamic toxicological profile. Toxicity appears to be strongly influenced by environmental factors, is mainly associated with liver tissue, and may involve different toxin groups depending on the period. These findings highlight the need for continued monitoring and for analytical approaches capable of capturing this complexity and assessing potential risks to public health.

## 4. Materials and Methods

### 4.1. Fish Collection, Identification, Measurements and Sample Extraction

Specimens of the pufferfish *Lagocephalus lagocephalus* were collected in Atlantic waters around Tenerife Island (Canary Islands, Spain) from two coastal locations. The first collection (*n* = 21) was conducted in March 2017 near the coast of Los Cristianos using purse seine fishing. A second batch of specimens (*n* = 22) were captured trammel fishing near the Candelaria coast, in depths between 20 and 50 m in November 2017. All fish were identified as *L. lagocephalus* in the Marine Science Departmental Unit of the University of La Laguna by biologist experts according to the morphological characteristics reported by Matsuura [2] and with the aid of fish guide pictures [6]. Measurements of total length (March: 36.5 ± 1.4 cm, November = 34.5 ± 2.4 cm) and weight (March: 389.7 ± 112.4 g, November = 385.8 ± 111.2 g), were also conducted for all individuals. After identification and conducting of measurements, a sample of liver, kidney, gonads, skin and muscle of each fish was carefully extracted. Samples were kept frozen at −20 °C until the selection of specimens for toxicological analysis.

### 4.2. Sample Preparation and Toxin Extraction

Toxin analyses were performed on organs from 15 specimens collected in March 2017 (length: 36.7 ± 1.5 cm; weight: 403.9 ± 120.4 g) and 12 specimens collected in November 2017 (length: 34.5 ± 3.2 cm; weight: 390.3 ± 136.4 g). These sub-samples were selected to represent the full biometric range (length and weight) of the total population collected in each event, ensuring the representativeness of the toxicological findings. Following the MBA screening, only those extracts that tested positive and maintained sufficient remaining volume were selected for further chemical characterization by UHPLC-MS/MS. Toxicity of the different organs was determined according to the method described by Kawabata [25]. For the toxin extraction, approximately 5 g of each organ (liver, kidney, gonads, skin, and muscle) was solubilized in 10 mL of 0.1% acetic acid (Riedel de Haën, Sigma–Aldrich, Seelze, Germany) and homogenized using Ultra-turrax homogenizer (Silverson L4RT, Silverson Machines Ltd., Buckinghamshire, UK) at 10,000 rpm for 15 min. After that, the solution was heated at 75 °C for 30 min in a boiling water bath, with occasional stirring. Then, the mixture was cooled and centrifuged at 5000 rpm for 10 min at 4 °C. The residue was re-extracted with an additional 10 mL of 0.1% acetic acid following the same heating, cooling, and centrifugation protocol. The combined supernatants were defatted by adding 10 mL of n-hexane, followed by stirring and centrifugation at 5000 rpm for 10 min at 4 °C. After phase separation, the aqueous phase (20 mL) was collected and filtered through a 0.45 μm membrane filter (Millipore, Burlington, MA, USA). The filtrate was then diluted with 0.1% acetic acid to a final volume of 25 mL. When less than 5 g of tissue was available, all reagent volumes were adjusted proportionally.

### 4.3. Mouse Biological Assay (MBA)

The mouse biological assay (MBA) was used to evaluate and quantify the toxicity of extracted samples based on the dose–death time relationship described by Kawabata (1978) [25]. An aliquot of 1 mL of each extracted solution was intraperitoneally injected into three male Albino Swiss mice (weighing between 19 g and 21 g), obtained from the Estabulario-Animalario, Servicio General de Apoyo a la Investigación (SEGAI), from Universidad de La Laguna (ULL). Animals were continuously observed, and the time to death was recorded. The neurotoxicity of the extracts was confirmed by the death of the mice after intraperitoneal injection. The time to death (i.e., the interval between injection and death) was recorded for each specimen and is detailed in Supplementary Table S1. This biological screening served to validate the presence of toxins and to select the most potent samples for subsequent chemical characterization via UHPLC-MS/MS. All animal procedures were conducted under the supervision of the personnel of the Animal Department of University of La Laguna, in accordance with EU Directives (Directive 2010/63/EU of the European Parliament and of the Council of 22 September 2010 on the protection of animals used for scientific purposes). Samples (1 mL) of 0.1% acetic acid heated for 30 min in boiling water were used as blank reference in this MBA analysis. At the end of the experiments, the animals were sacrificed using the rules of animal protection and ethical protocols of the Universidad de La Laguna (ULL) [reference number ULL-CEIBA2020–0397].

### 4.4. Chemicals and Certified Materials

Certified reference material containing tetrodotoxin (TTX, CAS 4368-28-9), 4,9-anhydro-TTX (4,9-anh-TTX, CAS 13072-89-4), and 4-epi-TTX (CAS 98242-82-1) in 1 mM acetic acid in water (pH 3.91) were supplied by CIFGA standards (Lugo, Spain). Certified concentrations of analytes were 21.0 (±1.3), 5.44 (±0.40), and 1.67 (±0.15) mg/kg, respectively. Daily working solutions were prepared by dilution in 0.1% (*v*/*v*) acetic acid in water. All solutions were stored in the dark at −18 °C.

LC-MS-grade acetonitrile (ACN) was obtained from Merck (Darmstadt, Germany), and deionized water was obtained from a Milli-Q system A10 (Millipore, Burlington, MA, USA). Formic acid (>98%) was from Honeywell (Jersey City, NJ, USA). Ammonium hydroxide solution for trace analysis (≥25% NH_3_ in water) and reagent-grade glacial acetic acid were from Sigma-Aldrich (Madrid, Spain).

### 4.5. Toxin Analysis by UHPLC-QqQ-MS/MS

The analyses were carried out in a Waters Acquity UPLC^®^ H-Class, consisting of an autosampler with a flow-through needle system and a quaternary pump from Waters Chromatography (Milford, MA, USA). The UHPLC system was hyphenated with an MS Xevo TQD (Waters Chromatography, Milford, MA, USA) with an electrospray ionization interface in positive and negative mode. Masslynx™ software (version 4.1) from Waters Chromatography was used to control the pumps and sample manager, as well as MS parameters and the collection and processing of spectrum data. Separation was performed on an Anionic Polar Pesticide column (130 Å, 5 µm, 2.1 mm × 100 mm) using a pre-column with the same stationary phase (5 mm × 2.1 mm, 5 µm), both from Waters Chromatography. The column and pre-column temperatures were set at 50 °C. The stationary phase as based on the ethylene bridged hybrid (BEH) particle with diethylamine groups operating under hydrophilic interaction liquid chromatography (HILC)–weak anion-exchange chromatography (WAC) conditions.

The separation method was adapted from Boundy et al. [22] with several modifications. The mobile phase consisted of water (solvent A) containing 0.03% (*v*/*v*) of formic acid and 0.06% (*v*/*v*) of NH_4_OH, and ACN (solvent B) containing 0.1% (*v*/*v*) of formic acid. The composition was initially set at 2/98 (*v*/*v*) A/B and maintained for 5.0 min at a flow rate of 0.4 mL/min. Then, it was changed to 50/50 (*v*/*v*), A/B in 2.5 min. The mobile phase composition was then held for 2.5 min while the flow rate was increased to 0.5 mL/min. The column was re-equilibrated first, returning to a composition of 5/95 (*v*/*v*), A/B, in 0.5 min. Later it was changed to 2/98 (*v*/*v*), A/B while increasing the flow rate to 0.7 mL/min in 0.3 min and maintained for 0.8 min. Finally, the initial flow rate of 0.4 mL/min was re-established in 0.4 min. The injection volume was 5 µL at 10 °C.

The MS analysis was performed in multiple reaction monitoring mode using the retention time and two different transitions as identification points [20]. The source conditions were as follows: capillary voltage 3.5 kV, source temperature 150 °C, desolvation temperature 600 °C, cone gas (N_2_) flow rate 150 L/h, desolvation gas (N_2_) flow 1000 L/h, collision gas (Ar) pressure 0.5 bar. Acquired analytical standards of TTX, 4,9-anh-TTX and 4-epi-TTX were used to experimentally establish the most intense MS/MS transitions in multiple reaction monitoring mode, whereas the rest of the analytes, i.e., STX, dcSTX, doSTX, NEO, dcNEO, GTX1, GTX2, GTX3, GTX4, GTX5, GTX6, dcGTX1, dcGTX2, dcGTX3, dcGTX4, C1, C2, C3, C4, M1, M2, M3 and M4, were identified using reported bibliography information. The multiple reaction monitoring transitions, cone voltage, and collision energies applied for each analyte are shown in Table S2.

## 5. Conclusions

The anomalous presence of *Lagocephalus lagocephalus* along the coastal waters of the Canary Islands during 2017 highlights a relevant and emerging toxicological concern. The results of this study demonstrate that toxicity in this species is not constant but temporally variable, affecting a substantial proportion of specimens and being predominantly associated with liver tissue.

A clear seasonal variability in the toxicological profile was observed. Specimens collected during the March 2017 episode showed a mixed toxicological profile consisting of both TTXs and PSTs, whereas those from the November 2017 episode were primarily characterized by the presence of saxitoxins (STXs) and their congeners. This shift in dominant toxin groups indicates that environmental and ecological factors play a decisive role in toxin accumulation and/or transformation within the species.

The use of optimized UHPLC–MS/MS methods enabled the identification of a broad spectrum of marine toxins, revealing the complex and dynamic nature of the toxicological profile of *L. lagocephalus* in Canary Island waters. These findings underscore the importance of applying advanced multi-toxin analytical approaches to adequately characterize potential hazards.

Overall, the variability and complexity of the toxic profiles observed reinforce the need for continuous and systematic monitoring programs. Given the potential implications for public health, particularly in regions where this species may be encountered or consumed, sustained surveillance and risk assessment strategies are essential to mitigate health risks associated with toxic pufferfish species.

## Figures and Tables

**Table 1:** UHPLC-QqQ-MS/MS toxicological qualitative profile (presence and absence) of tetrodotoxins and paralytic shellfish toxins in toxic liver of *Lagocephalus lagocephalus*.

Specimen ID	Sampling Event	TTX	4-epi-TTX	STX	neoSTX	dcSXT	dcneoSTX	doSTX	M2
01	Mar 2017	x	x					x	x
02	Mar 2017	x	x				x	x	
16	Mar 2017					x	x		
20	Mar 2017		x			x	x	x	
10	Nov 2017	x		x	x	x	x	x	
15	Nov 2017			x		x	x	x	
21	Nov 2017			x	x	x	x		

Only toxic specimens with sufficient extract volume remaining after MBA screening were included in the UHPLC-MS/MS analysis.

## Data Availability

Data is contained within the article or Supplementary Material.

## Supplementary Materials

The following supporting information can be downloaded at: https://www.mdpi.com/article/10.3390/md24060195/s1, Table S1: Mouse biological assay (MBA) results for liver extracts of *Lagocephalus lagocephalus*. Samples from the March 2017 event are highlighted in blue, and samples from November 2017 are highlighted in green; Table S2: Multiple Reaction Monitoring (MRM) conditions and transitions for the determination of target analytes; Figure S1: Multiple Reaction Monitoring chromatograms of TTX, PST and its analogues detected in liver samples of *Lagocephalus lagocephalus*.
