# Supplementary material for: Toxicity Profile of the Oceanic Pufferfish Lagocephalus lagocephalus in the Eastern Atlantic Area
Source: Mar Drugs. 2026 Jun 1;24(6):195. doi: 10.3390/md24060195 (PMC13301243; doi:10.3390/md24060195)
Supplement: Supplementary file 1 [file marinedrugs-24-00195-s001.zip › marinedrugs-4288838-supplementary.pdf]

Supplementary Material

Toxicity profile of the oceanic pufferfish *Lagocephalus lagocephalus* in the eastern Atlantic area

Nathália Nocchi[1,2], Álvaro Santana Mayor[3], Adrián Conde Díaz[3], Víctor Hernández-Lopez[1], Adriana Rodríguez Hernández[4], Alberto Brito[4], Ana R. Díaz-Marrero[2,5], José J. Fernández[1,2,6]

[1] Instituto Universitario de Bio-Orgánica Antonio González (IUBO AG), Universidad de La Laguna (ULL), Avenida Astrofísico Francisco Sánchez 2, 38206 La Laguna, Tenerife, Spain; nathalianocchi@ull.edu.es; jjfercas@ull.edu.es
[2] Biotecnología Marina, IUBO-ULL, Unidad Asociada al IPNA-CSIC, 38206 La Laguna, Spain
[3] Departamento de Química, Unidad Departamental de Química Analítica, Facultad de Ciencias, Universidad de La Laguna (ULL). Avenida Astrofísico Fco. Sánchez 3, 38206 La Laguna, Spain; asan-tanm@ull.edu.es; acondedi@ull.edu.es; asantanm@ull.edu.es; acondedi@ull.edu.es
[4] BIOECOMAC (Biodiversidad, Ecología Marina y Conservación). Departamento de Biología Animal, Edafología y Geología, UD Ciencias Marinas, Facultad de Ciencias (Sección Biología), Universidad de La Laguna (ULL), Avenida Astrofísico Fco. Sánchez 3, 38206 La Laguna, Spain; adrianar@ull.edu.es; abrito@ull.es
[5] Instituto de Productos Naturales y Agrobiología (IPNA), Consejo Superior de Investigaciones Científicas (CSIC), Avenida Astrofísico Francisco Sánchez 3, 38206 La Laguna, Tenerife, Spain; adiazmar@ipna.csic.es
[6] Departamento de Química Orgánica, Universidad de La Laguna (ULL), Avenida Astrofísico Francisco Sánchez 3, 38206 La Laguna, Tenerife, Spain

Table S1. Mouse Biological Assay (MBA) results for liver extracts of *Lagocephalus lagocephalus*. Samples from the March 2017 event are highlighted in blue, and samples from November 2017 are highlighted in green.

Specimen ID	Sampling event	Length (cm)	Weight (g)	Death time (min)
**01**	**Mar 2017**	**35.0**	**352.0**	**13.0**
**02**	**Mar 2017**	**36.5**	**496.0**	**9.5**
03	Mar 2017	38.0	345.0	ND
04	Mar 2017	38.2	386.4	ND
05	Mar 2017	37.0	400.8	ND
12	Mar 2017	40.0	806.0	ND
13	Mar 2017	35.0	404.4	ND
14	Mar 2017	36.0	387.4	6.0
15	Mar 2017	37.0	360.6	ND
16	Mar 2017	38.2	363.2	10
17	Mar 2017	35.2	362.0	ND
18	Mar 2017	37.0	273.2	ND
19	Mar 2017	35.0	395.8	ND
20	Mar 2017	37.0	350.4	8.5
21	Mar 2017	35.0	376.0	ND
04	Nov 2017	34.0	386.4	ND
08	Nov 2017	32.5	341.0	ND
10	Nov 2017	33.5	322.6	16.0
12	Nov 2017	44.2	806.0	17.0
13	Nov 2017	34.1	404.4	ND
15	Nov 2017	35.5	360.6	4.0
17	Nov 2017	35.2	362.0	ND
18	Nov 2017	32.6	273.2	10.0
19	Nov 2017	33.7	395.2	ND
20	Nov 2017	32.6	350.4	ND
21	Nov 2017	33.1	376.0	7.0
22	Nov 2017	33.0	304.6	ND

ND = Not detected death in the first 24 hours.

Table S2. Multiple Reaction Monitoring (MRM) conditions and transitions for the determination of target analytes.

Analyte	Ionization mode	MRM transitions *(m/z)*	Cone voltage (V)	Collision energy (eV)
TTX/4-epi-TTX	ESI+	320.1 > 301.9	46	24
	ESI+	320.1 > 161.9	46	38
4,9-Anh-TTX	ESI+	302.2 > 161.9	66	36
	ESI+	302.2 > 59.9	66	32
STX	ESI+	300.1 > 204.1	10	23
	ESI+	300.1 > 138.0	10	30
dcSTX	ESI+	257.1 > 126.1	10	19
	ESI+	257.1 > 222.0	10	22
doSTX	ESI+	241.1 > 60.0	10	23
	ESI+	241.1 > 206.1	10	22
NEO	ESI+	316.1 > 126.0	10	26
	ESI+	316.1 > 220.1	10	23
dcNEO	ESI+	273.1 > 126.1	10	20
	ESI+	273.1 > 225.10	10	18
GTX1	ESI-	410.1 > 367.1	10	15
	ESI-	410.1 > 349.1	10	22
GTX2	ESI-	394.1 > 351.1	10	16
GTX3	ESI+	396.1 > 298.1	10	17
	ESI-	394.1 > 351.1	10	16
GTX4	ESI+	412.1 > 314.1	10	18
	ESI-	410.1 > 367.1	10	15
GTX5	ESI+	380.1 > 300.1	10	16
	ESI-	378.1 > 122.0	10	25
GTX6	ESI+	396.1 > 316.1	10	15
	ESI-	394.1 > 122.0	10	25
dcGTX1	ESI-	367.1 > 274.1	10	20
	ESI-	367.1 > 349.1	10	17
dcGTX2	ESI-	351.1 > 164.0	10	30
	ESI-	351.1 > 333.1	10	17
dcGTX3	ESI+	353.1 > 255.1	10	18
	ESI-	351.1 > 333.1	10	17
dcGTX4	ESI+	369.1 > 271.1	10	18
	ESI-	367.1 > 349.1	10	17
C1	ESI-	474.1 > 122.0	10	30
	ESI-	474.1 > 351.0	10	25
C2	ESI+	396.1 > 298.0	18	20
	ESI-	474.1 > 122.0	10	30
C3	ESI+	412.1 > 332.1	18	16
	ESI-	490.1 > 410.1	10	20
C4	ESI+	412.1 > 314.0	18	20
	ESI-	490.1 > 392.1	10	20
M1	ESI+	396.1 > 316.0	10	20
	ESI+	316.1 > 148.0	10	20

M2	ESI+	316.1 > 298.1	10	15
	ESI+	316.1 > 220.1	10	18
M3	ESI+	412.1 > 332.0	10	10
	ESI+	332.1 > 314.1	10	20
M4	ESI+	332.1 > 314.1	10	20
	ESI+	332.1 > 108.0	10	30

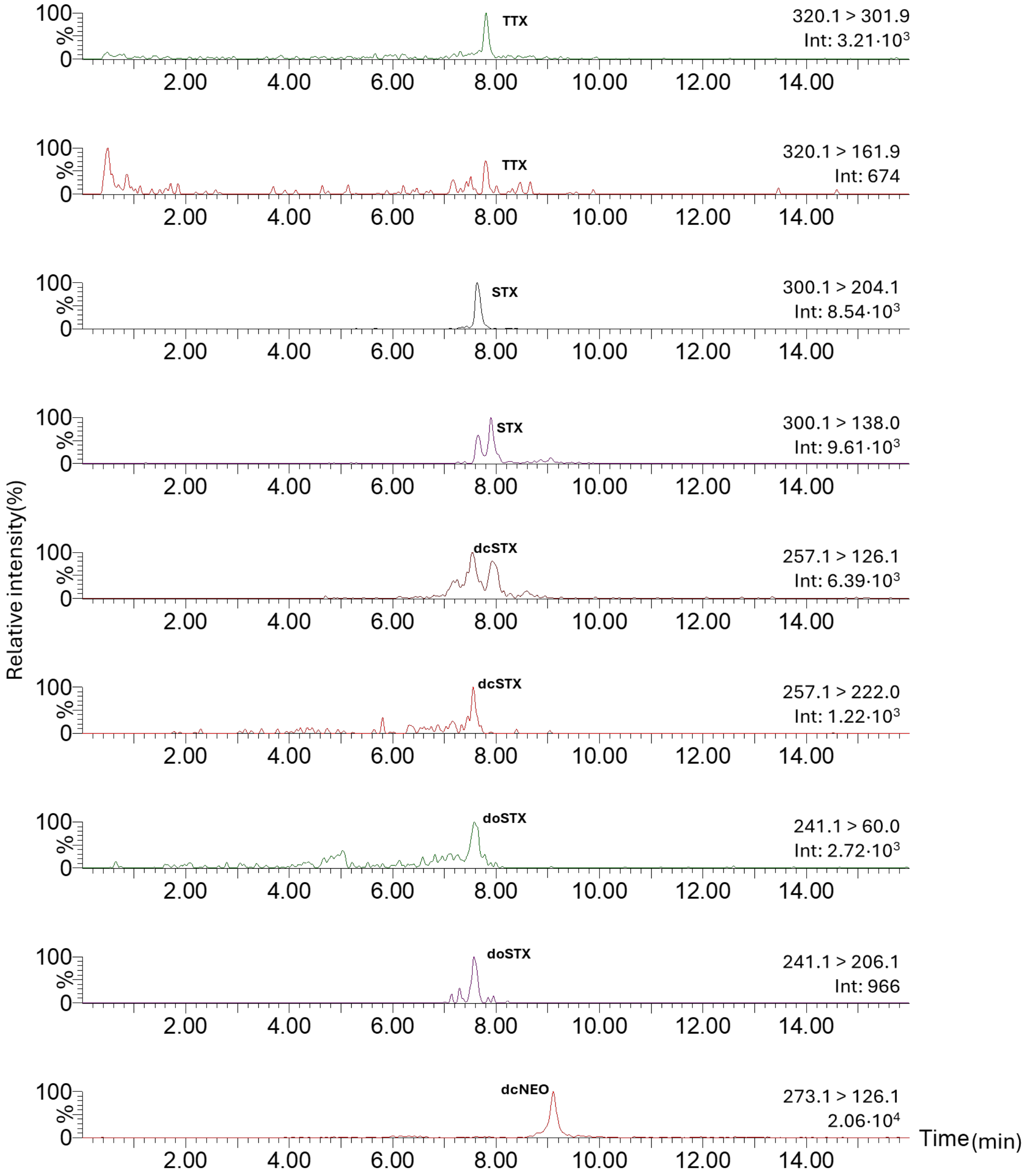

Figure S1. Multiple Reaction Monitoring chromatograms of some TTX, PST and its analogues detected in liver samples of *Lagocephalus lagocephalus*.
